# “*We want to know the cause of neonate's death to prevent similar incident in the future* …”: A Formative Study to Introduce Minimally Invasive Tissue Sampling Procedure at Community Level in Butajira

**DOI:** 10.4314/ejhs.v34i5.2

**Published:** 2024-09

**Authors:** Mirgissa Kaba, Kalkidan Solomon, Tesfamichael Awoke, Tewodros Yalew, Amha Mekasha, Lulu Muhe

**Affiliations:** 1 School of Public Health, Addis Ababa University; 2 Department of Pathology, School of Medicine, Addis Ababa University; 3 Department of Paediatrics, School of Medicine, Addis Ababa University

**Keywords:** newborn death, neonatal health, formative research, MITS, Butajira

## Abstract

**Background:**

Diagnostic autopsy has been in use for long to determine the cause of death. Since recently however ‘Minimally Invasive Tissue Sampling’ (MITS) is introduced to determine definitive cause of neonatal death. This study describes locally established facilitators to introduce MITS procedures to determine cause of neonatal death in Ethiopia.

**Methods:**

Exploratory study was conducted in Butajira community where twenty-two key informants representing community opinion leaders' health care workers, five in depth interviews with parents who recently lost neonates and eight FGDs with community members were completed to generate evidences in line with the research question. Interviews and discussions were audio recorded, transcribed verbatim and analysed facilitated by open-code software. Thematic analysis was applied to identify and interpret patterns of the evidences

**Results:**

In Butajira, ANC and delivery in health facilities was found to have improved over the years. Yet, child death remains an outstanding problem. While different factors were identified to cause the death of a child, relatively few participants choose to accept newborn death as a natural occurrence or will of the creator. Majority of the study participants expressed interest to know definitive cause of death using MITS. Yet, awareness about MITS and how it works was unanimously desired. It was found that husbands and wives are key to authorize MITS procedure while community opinion leaders including religious leaders were identified as key to influence parental decisions for the procedure

**Conclusion:**

Building awareness of the community members and engagement of opinion leaders is critical to introduce MITS.

## Introduction

Ethiopia has shown remarkable progress in child health and survival (1). Despite this, still many children in Ethiopia die before getting appropriate support at health facilities. This has to do with various factors at parents, community, and facility levels (2, 3) contributing to limited understanding of the causes of death poorly informed public health interventions to control death of neonates (1). Achievement of United Nations' Sustainable Development Goals for good health and well-being, as well as elimination of preventable newborn and child deaths calls to determine the definite causes of stillbirths and neonatal deaths (4).

In developed countries, the gold standard to establish the causes of death is the complete diagnostic autopsy. However, this is not a regular function in resource-poor settings like in Ethiopia (5). In addition to the limited level of accuracy in determining the causes of death, verbal autopsy is not widely used due to poor health infrastructure, limited competence of health care providers, and religious or cultural apprehension for the practice of post-mortem evaluation (6).

The most troubling deaths are those for which there are no clear signs and symptoms on the one hand and difficulty to assume the cause of death. Given this, it remains critical to develop feasible and more direct methods to determine definitive cause of death. To circumvent such problems, the WHO recommends the use of non-invasive indirect methods including verbal autopsy (7, 8). In line with such efforts to develop approaches and methods, in recent years, minimal invasive autopsy (MITS) procedure has been introduced and applied as an alternative to traditional diagnostic autopsy (9, 10). Although experiences with such techniques is limited, evidences reveal that this technique is feasible to determine the cause of neonatal and infant death (7, 10, 11)

The practice of MITS in a resource-limited setting would offer authentic evidence on the cause of the child's death, reduce uncertainties regarding causes of death and offer concrete evidence to inform public health interventions in developing countries (11, 12). Implementation of the new MITS procedure requires prior understanding of the cultural and religious norms and practices at the community level to determine the feasibility of introducing the MITS procedure (1,13). Few studies documented the detrimental role of the perception and attitude of community leaders, members of the community, spouses, and healthcare professionals t in introducing the MITS procedure at the community level (1, 14, 15). Yet, the sociocultural, religious, and behavioural factors in different contexts may offer different approaches and processes to introduce MITS at the community level (12, 16). This formative study aims to generate a culturally sensitive methodological approach to inform the introduction of the MITS procedure in Butajira, Southern Ethiopia.

## Methods

**Study setting**: The study was conducted in three districts of Health and Demographic Surveillance Site (HDSS) located in East Gurage Zone of Central Ethiopian Regional State, approximately 1380 kms south of Addis Ababa. Ten Kebeles, the smallest administrative unit in Ethiopia, of Butajira district, has served as the demographic and health survey hub of the School of Public Health of Addis Ababa University since 1986. The Butajira DHSS documents vital events such as death, pregnancies, and births every 3 months (17)^1^.

The climate varies from dry lowland areas at altitudes around 1,500m (tropical climate) to cool mountainous areas up to 3,500 m above mean sea level (temperate climate). The total population in the district is estimated at 76,350 in 2015.

**Study design**: Explorative qualitative research was conducted in rural and urban kebeles of Butajira HDSS.

**Study population**: Community opinion leaders, health care workers public health practitioners, parents of index neonates, and community members (residents in the community who have children) participated in the study. The number of participants was determined based on the saturation of evidence at each level.

**Data collection**: Data was collected using in-depth and key informant interviews as well as focus group discussions (FGD). A total of 22 key informant interviews with community leaders including influential leaders in the community as well as health care providers including Health extension workers (HEWs); five in-depth interviews with parents of recently deceased child and eight FGDs with a total of sixty-four men and women members of the community with live children were completed.

Participants were engaged in active discussions to share personal and shared experiences and evidence on local values of pregnancies, problems associated with neonatal illnesses and death (stillbirths, death of new-borns, perceived causes of death), local interest to know the definitive cause of death (using needle punctures after death), processes of burial management and care for the family, locally viable and sound strategy to obtain consent for MITS procedure, and preferred setting to conduct MITS (home/Health Facilities).

Data were collected by trained health professionals (health officers and Nurses who have Mph) with proven experience in qualitative research undertakings with close supervision during data collection. Interviews and discussions were audio recorded while scribbles were taken and translated into field notes.

**Data analysis**: The audio-recorded data were transcribed verbatim, translated to English, and uploaded on to Open code software for coding. Thematic analysis was applied where themes and sub-themes were inductively developed. Two of the researchers (TY and KS) read and re-read the transcripts and developed codes independently which were then checked by the senior researcher MK who reviewed, refined and approved the codes. Codes were brought together to develop categories that were further conceptualized into themes for interpretation and presentation without affecting the original meanings of the evidence. Verbal quotes that represent common arguments were presented to substantiate the findings.

**Data quality assurance**: Research assistants were recruited based on their prior qualitative research experience. Extensive training was provided before the commencement of data collection to help research assistants internalize the essence of the study, objectives, tools, and ethical elements. During data collection, at the end of every day, peer debriefing was held to among others ensure the consistency of data. After data collection was completed, transcripts were read re-read, and coded by two of the research team members which was checked for consistency and verified by. Data were triangulated by method, place, and individual participant's profile. Effort was made to maintain the original meanings when interpreting the data.

**Ethical consideration**: The study protocol was approved by the IRB of the College of Health Sciences, Addis Ababa University with a ref, no 016/20/pedi. During the data collection, participants were provided comprehensive information about the purpose of the study, implications, and rights to participate is solely within the discretion of individuals recruited for the study.

## Results

A total of 64 male and female community members with live children took part in eight FGD sessions, 22 key informant interviews with opinion and religious leaders and five parents who recently lost neonates participated in the study. Findings were summarized under major themes of perceived value for pregnancy and child birth, perceived cause of death, interest to know cause of death, preferred place for MITS procedure and authority to consent for MITS procedure.

**Perceived value for pregnancy and child birth**: Irrespective of their characteristics and number of children born to the family, participants explained that, women and the family members consider every pregnancy as an important event and accomplishment for a woman and the family. Although, women participants reported to have fairly good awareness about family planning, the need for more children remains unlimited. Woman considers pregnancy as great gift and source of recognition and respect in the community. For husband, it is considered as a gift of the Almighty and useful contribution to his kins. Nearly, all participants argued that woman breaks the news of her pregnancy to her husband while family members and close friends will know about the pregnancy as it reveals itself. Women explained that signs of pregnancy includes failure of menstruation cycle, nausea, and loss of appetite, change in mood, body pains like fever and back pain. In order to confirm pregnancy and start Antenatal care (ANC), Health Extension Workers (HEWs) encourage women to visit health centers.

*“When the husband hears the news of his wife's pregnancy, he gets happy since this helps him meet his responsibility to contribute to his bloodline” (Community elder, KII, Misrak Meskan)*.

*“Although the reaction of husbands varies on the wife's visit to the health center in connection to ANC, all husbands are happy when their wife gets pregnant. My husband was very happy and advised me to get a check-up at a health center (Woman, FGD, Wurib)”*

All participants argued that ‘children are considered as blessing and sign of good luck’. However, usually, women lack adequate support from husbands and family members during their pregnancy. Husbands and family members role in connection to women's pregnancy was not clear. However, as primary care provider to the family, husbands tent to focus on ensuring the livelihood of the family. Support from community members and religious and community leaders usually is limited to visit the woman after she delivers. Even that was found to depend on women's social network in the community.

*“In our community, support, and care to women from community members depends on the level of support and her social network within the community” (Men, FGD, K04)*

**Perceived cause of neonatal death**: Despite the celebration of pregnancy and childbirth by family members and the positive value of it in the community, participants shared their concern and shock at the death of a neonate.

Responses to ‘What are the causes of such death’ reveal that missing food a pregnant woman have craved for, workload including carrying heavy stuff and failure to attend ANC services during pregnancy; and poor handling of the newborn, exposure to cold, failure of the newborn to breastfeed, diseases and traditional practice like feeding butter after delivery were identified wide range of cause of early death of a child. Nonetheless, all participants attributed death ultimately to God's will.

*“The death of a neonate is God's decision. Yet, lack of care for women during pregnancy and failure to care for the mother and newborn after delivery facilitates the death of a neonate (KII religious leader)*

Around two out of three participants argue that, nowadays most of the pregnant woman attends health facility in connection to her pregnancy. Yet, still women tend to deliver at home that may result in early death of children.

*Children may die if the baby is not born in health facility and is not taken to health facility when sick. Often when baby gets sick efforts are made to treat them at home which may facilitate neonatal death. (Male, FGD, Misrak Meskan)*

**Interest to know cause of death**: Religious leaders, elderly members of the community and husbands were found to share common opinion on the need to know the cause of neonate's death. Thy were found to attribute cause of death to the will of God and failure to care for mother and the new born. As a result, they are not interested to know the cause of death. On the other hand, women, young parents and participants in urban settings have expressed interest to know the cause of death.

*Mothers want to know the cause of death. They ask for causes and discuss with neighbours on what could be the cause of death. One of those contemplates if the contraceptive she uses has caused death of the newborn. They are worried if they can ever get another baby after this?” (HEW, IGI, Shershera Bido)*. Woman participant were found to share common interest to know the cause of death; *“We want to know the cause of neonate's death to prevent similar incident in the future and also advice other women in the neighbourhood” (Woman, FGD1, Misrak Meskan)*. Based on her extensive engagement with women, a HEW argued that knowing cause of death helps parents to understand what went wrong and avoid similar incident in the future. *“The reason on why they want to know the reason of death is to prevent similar incident in the future in the family.” (HEW, KII, K04)*

**Preference of place for MITS procedure**: Finding shows that burial of the neonate takes place at the family's backyard. The burial event is facilitated and attended only by close family members and close neighbours. The grief is not taken as seriously as the death of the elderly. The question of the preferred place of MITS procedure reveals that all those who are interested to know the cause of death needed to know details of how MITS works. As few as one in six women and HEWs suggested providing relevant information on MITS to women during ANC follow-up would be useful. *“Provision of more information about MITS, how it works and its implications may facilitate the introduction of MITS in the community to determine definitive cause of death. Such information provision could be organized at the community as well as at health facility levels”* (HCP, HC). In addition, interest was vivid to gain knowledge on MITS, which is grossly unknown in the community and its procedure. *“We haven't heard about MITS before. What you told me about it is very interesting and I appreciate it. But a woman can't make the decision alone to allow the procedure. So, every community member should be educated on MITS procedure and its benefits” (Woman, IDI, K04)*.

However, one out of four women participants and about one out of three male participants expressed concern and fear about what health professionals may do to the remains of the body. *“First of all, if doctors use MITS as an opportunity to use the corpse for practice, we feel quite embarrassed. But if it is for saving lives at least in the future, we will be happy to see MITS implementation”. (Woman, KII, Wurib)*.

Participants argued that the place where the MITS procedure takes place is determined by the place of the neonate's death. If the neonate dies in the health facility, a procedure is suggested to take place within the facility. If death occurs at home, it was argued that moving the remains to a health facility is unfavourable and that the procedure should take place at home. Participants invariably contended that most neonatal death occurs at home and that procedure should take place within the household.

*“I suggest the MITS procedure should take place where death occurs. Since most death occurs at home, it is not culturally acceptable to take the remains of a neonate to the hospital for examination. Health professionals should come to the place of death to take samples.” (Male, KII, K04)*.

**Consent for MITS procedure**: The approach to seeking consent reveals diverse views. About three out of four participants irrespective of profile argued that verbal explanation could serve the purpose of getting consent. Claiming that showing a photo of a child could be disturbing but very few have suggested to use of an illustrative explanation of the procedure to get consent.

*Request for consent should be made convincingly with implications. Provision of prior information about MITS at different levels could facilitates the consenting process. Certainly, giving consent with such an explanation would make it. (Woman, IDI, K04)*

In as much as the place where the neonate dies determine where the procedure could take place, it was argued that this will determine who should give consent for the procedure. If the child dies at health facility, the person who is at the facility when the child dies, which is likely to be the parents or close kin makes the decision for MITS procedure. If the child dies at home, the decision is made ultimately by father or designated local leader particularly closer kins in the presence of the health professional. *“The one that should be asked for permission should be the parent. More particularly husband, as head of the household, is the person that makes the decision to launch MITS” (woman, FGD, Wurib)*. It was unanimously argued that influential local leaders including religious leaders could facilitate the consenting process given they are well aware of the procedure.

## Discussion

Verbal autopsy has for long been the known technique to determine cause of death although it is not the most reliable one. Application of minimally invasive tissue sampling (MITS), has become a new procedure to determine the definitive cause of death especially for neonates (18). Ethiopia, as a country recognized for its cultural mosaic and consequent diverse disease discourses, the cause of death generally remains to vary. Trauma/injury, complications in labour, lack of proper care and hygiene, exposure to cold and/or the will of the creator are often identified as causes of death including children's death (12, 19).

The Study clarified the interest to know more about MITS procedure to determine cause of death. The interest to introduce the procedure thus depends on how well the public is aware of MITS and its procedure. Building trust in the process leading to MITS was highlighted as a major success factor. The role of religious and community leaders as influencers in the community was emphasized as helpful in facilitating consent for the procedure. This finding contends with the suggestion by the Child Health and Mortality Prevention Surveillance (CHAMPS) network where community influential leaders were reported to play a detrimental role in facilitating the implementation of the procedure at the community level (12, 14, 20).

This finding reveals that husbands have the ultimate decision-making authority to apply the procedure. Similar studies from Nigeria and Tanzania reveal the father as an authoritative figure (14, 15) who decides to implement MITS. A study from Bangladesh, a Muslim-dominant community, showed that consent granted by the husband or the community leaders is considered rational and final (4). Although further study may help to chart how to facilitate MITS understanding at the community level, the potential alignment of educational intervention with routine maternal health intervention at the community level could be a potential opportunity with routine community-level maternal and child health interventions.

On the place where the MITS procedure could take place, participants would like the procedure to take place at the household level when the newborn dies at home while it could happen at a health facility if the newborn dies at the facility. A recent study from Ethiopia suggested that with standardized training and supportive supervision, nurses could manage the MITS procedure at the home where the neonate dies (21).

The application of minimally invasive tissue sampling (MITS) is an important procedure, particularly in Lower-and Middle-Income Countries to determine definitive causes of neonate death thereby improving the health of children. While including MITS education into routine health education activities at community and facility levels would improve public awareness about the procedure and implications, how best this could roll requires further study. Community influential leaders were found to play an important role in facilitating the implementation of MITS procedures while fathers/husbands are the ultimate decision-makers. Strengthening outreach services by health professionals from the health facility is an important vehicle to build trust and improve relations between the community and the health system in addition to improving running MITS at the community level.

## Footnotes

1 Shamebo D, Sandström A, Wall S. The Butajira rural health project in Ethiopia: epidemiological surveillance for research and intervention in primary health care. Scand J Prim Health Care. 1992 Sep;10(3):198-205. doi: 10.3109/02813439209014061. PMID: 1410950
